# Increasing Charge Carrier Mobility through Modifications of Terminal Groups of Y6: A Theoretical Study

**DOI:** 10.3390/ijms24108610

**Published:** 2023-05-11

**Authors:** Yunjie Xiang, Chunlin Xu, Shaohui Zheng

**Affiliations:** 1 School of Materials and Energy, Southwest University, Chongqing 400715, China; 2 Chongqing Key Laboratory for Advanced Materials and Technologies of Clean Energies, Southwest University, Chongqing 400715, China

**Keywords:** non-fullerene acceptor, Y6, organic solar cell, photovoltaic property, terminal groups

## Abstract

The applications of non-fullerene acceptor Y6 with a new type of A_1_-DA_2_D-A_1_ framework and its derivatives have increased the power conversion efficiency (PCE) of organic solar cells (OSCs) up to 19%. Researchers have made various modifications of the donor unit, central/terminal acceptor unit, and side alkyl chains of Y6 to study the influences on the photovoltaic properties of OSCs based on them. However, up to now, the effect of changes of terminal acceptor parts of Y6 on the photovoltaic properties is not very clear. In the present work, we have designed four new acceptors—Y6-NO_2_, Y6-IN, Y6-ERHD, and Y6-CAO—with different terminal groups, which possess diverse electron-withdrawing ability. Computed results show that with the enhanced electron-withdrawing ability of the terminal group, the fundamental gaps become lower; thus, the wavelengths of the main absorption peaks of UV-Vis spectra red-shifts and total oscillator strength increase. Simultaneously, the electron mobility of Y6-NO_2_, Y6-IN, and Y6-CAO is about six, four, and four times faster than that of Y6, respectively. Overall, Y6-NO_2_ could be a potential NFA because of its longer intramolecular charge-transfer distance, stronger dipole moment, higher averaged ESP, enhanced spectrum, and faster electron mobility. This work provides a guideline for the future research on modification of Y6.

## 1. Introduction

During the past few years, the power conversion efficiency (PCE) of single-junction organic solar cells (OSCs) has been dramatically increased, and researchers have been creating new records. At present, the highest PCE of single-junction OSCs reaches 19% [1]. All these breakthroughs can be attributed to the emergence of the revolutionary Y6 (BTPTT-4F) non-fullerene acceptor (NFA), synthesized by Zou and co-workers [2]. With a new A_1_-DA_2_D-A_1_ (A: acceptor group; D: donor group) molecular framework, Y6 consists of two strongly electron-withdrawing peripheral groups linked by a ladder-like multi-fused DAD group. Compared with the previous A-D-A type of NFAs such as ITIC and its derivatives, the introduced DA_2_D core in Y6 provides a stronger intramolecular electronic push–pull (IEPP) effect. This can further reduce the optical gap and enhance light harvesting in the near-infrared (NIR) region [3]. So, the Y6-based OSC is superior to the ITIC-based one, although the latter’s PCE is already up to 13.0% [4].

Until now, researchers have applied several strategies to make molecular modifications of Y6. The first one is to change the side alkyl chain of Y6. For instance, the side alkyl chains on the pyrrole ring of Y6 were substituted with 2-butyloctyl chains to synthesize the new acceptor, BTP-4F-12 [5], and a higher PCE of 16.4% (OSC: PBDB-TF/BTP-4F-12) was achieved, whereas the PCE of the prototype of the PBDB-TF/Y6 OSC is 15.3%. Another example is NFA L8-BO, which was synthesized by substituting the alkyl chain on the thiophene ring of Y6 with branched alkyl chains (2-butyloctyl) [6]; the PM6/L8-BO-based ternary OSCs had a high PCE of 18.32%. The second modification strategy is the change of the donor unit of Y6. For example, by extending the π-conjugation length of Y6 by fusing one more thiophene onto one side of the thienothiophene unit, BP5T-4F was made [7], and the PCE of the OSC based on PM6/BP5T-4F reached 16.7%. Altering the central acceptor unit of Y6 is a third modification strategy. For example, the S atom on the central thiadiazole was replaced by an N atom, and an alkyl branch was attached to the N atom to make new molecule Y1-4F [8]; the PCE of the PBDB-TF/Y1-4F-based OSC was only 14.4%. Furthermore, the S atom on the central thiadiazole was replaced with an Se atom to obtain molecule Y6Se [9], and the OSC based on as-cast D18:Y6Se had a PCE of 17.7%, which is higher than that of the D18:Y6-based device (17.1%). The fourth modification strategy is to halogenate or expand the end groups of Y6. For instance, all fluorine atoms of Y6 were substituted with chlorine to obtain new NFA BTP-4Cl [10], and the PBDB-TF/BTP-4Cl-based OSC achieved a PCE of 16.5%, which was higher than the PCE (15.6%) of the PBDB-TF/Y6 OSC. Another example is BTP-FCl-FCl, in which there is one Cl substitution on each terminal group of Y6, and the PCE of the BTR-Cl/BPT-FCl-FCl OSC is 15.3% [11]. On the other hand, an asymmetric non-fullerene acceptor LL3, one of the Y6 derivatives, was designed and synthesized with one expanded norbornyl-modified 1,1-dicyanomethylene-3-indanone (CBIC) terminal group and a chlorinated 1,1-dicyanomethylene-3-indanone (IC-2Cl) terminal group, and the OSC based on LL3 had a PCE of 16.82%, which was higher than symmetric LL3-2Cl (14.4%) [12]. Another asymmetric NFA is BTP-S9, with two terminals (IC-2Cl and NC-2F (expanded ring)); the PM6:BTP-S9 OSC exhibited the efficiency of 17.56% [13]. As mentioned above, the modifications on alkyl chains, donor units, central acceptor units, and the end group of Y6 have been successfully conducted to investigate their effects on photovoltaic properties. Among them, the modification of end groups can change the stacking morphology and charge transport. The synthesis of modifying end groups is relative easy, and the stacking of terminal groups is of great significance [14]. So, it is particularly important. However, there is still a lack of research in this area, such as the substitution of terminal groups with non-halogen electron-withdrawing groups and the study of the substitution of entire end groups, which may have a significant impact on their photovoltaic performance.

**Figure 1:**
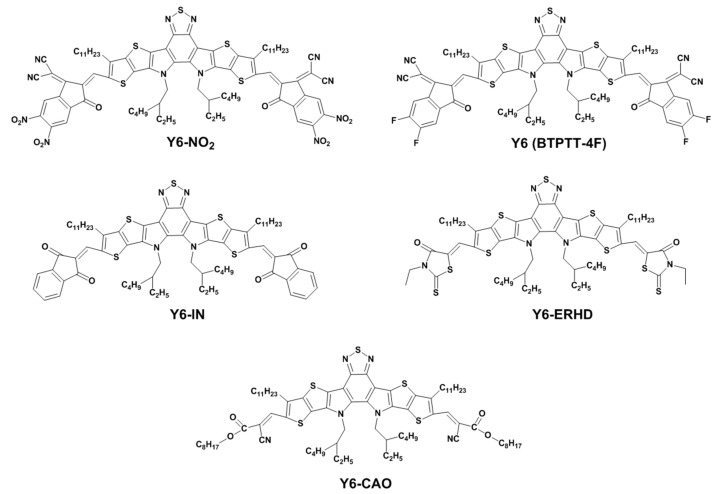
Chemical structures of Y6-NO_2_, Y6, Y6-IN, Y6-ERHD, and Y6-CAO. The electron-withdrawing ability of terminal group of these molecules is Y6-NO_2_ > Y6 > Y6-IN > Y6-ERHD > Y6-CAO.

Therefore, in the present work, we have designed and modeled a series of symmetric Y6 derivatives, i.e., Y6-NO_2_, Y6-IN, Y6-ERHD, and Y6-CAO, as shown in Figure 1. The electron-withdrawing ability of the terminal groups of these molecules is Y6-NO_2_ > Y6 > Y6-IN > Y6-ERHD > Y6-CAO. Density functional theory (DFT) [15] and time-dependent density functional theory (TDDFT) [16] are used to study these molecules because they have proved to be highly appropriate and efficient for calculations of electronic structures and excited states of organic molecules with proper XC kernels [17]. The effect of the changes in terminal acceptor parts on the dipole moment, FMO energy, UV-Vis absorption spectra, electrostatic potential (ESP), exciton binding energy, electron/hole distribution, and charge carrier mobility have been systematically studied.

The purposes of this work are two-fold:

(1) To investigate the effects of different terminal acceptor units on the photovoltaic properties of Y6.
(2) To find new promising acceptors (if possible).

## 2. Results and Discussions

### 2.1. Dipole Moments and Electrostatic Potential of the Modified Molecules

Dipole moment has important effects on molecular packing and charge separation in thin film [18]. As shown in Table 1, Y6-NO_2_, Y6, Y6-IN, Y6-ERHD, and Y6-CAO possess dipole moments of 15.56, 0.82, 4.16, 6.31, and 2.96 Debye, respectively. We therefore can conclude that the modification of the terminal groups has a great influence on their dipole moments. In particular, the strongest electron-withdrawing ability of the terminal groups of Y6-NO_2_ causes the longest distance between the positive and negative charge centers, thus Y6-NO_2_ has the largest dipole moment. Y6-IN, Y6-ERHD, and Y6-CAO have moderate dipole moments (4.16, 6.31, and 2.96 Debye, respectively), which are all larger than that of Y6. This should benefit crystallinity in thin film [19]. One good example is the BTP-4Cl, synthesized by Hou and co-workers [10]. It has a dipole moment of 1.44 Debye, which is slightly larger than that of Y6 (0.64 Debye). So, the crystallinity of BTP-4Cl is better than that of Y6, and the PCE of the OSC with BTP-4Cl as an NFA and PM6 as a donor rises to 16.1% [10]. On the contrary, another opposite example is BTPT-4F NFA (A_1_–DA_2_D–A_1_ type), which was synthesized by Fan et al. [7]. The PCE of a single-junction P2F-EHp/BTPT-4F OSC is just 1.09%. There exist two reasons for this sharp reduction: One is that the BTPT-4F molecule does not possess two C_11_H_23_ side alkyl chains and the other is that too large a dipole moment (12.34 Debye) results in strong aggregation and phase separation. Y6-NO_2_, possessing the largest dipole moment (15.56 Debye), may have strong aggregation and phase separation. In summary, on account of the similar structures and the same side alkyl chains, Y6-IN, Y6-ERHD, and Y6-CAO, with moderately larger dipole moments, may possess better performance than Y6 in OSCs. 

**Figure 2:**
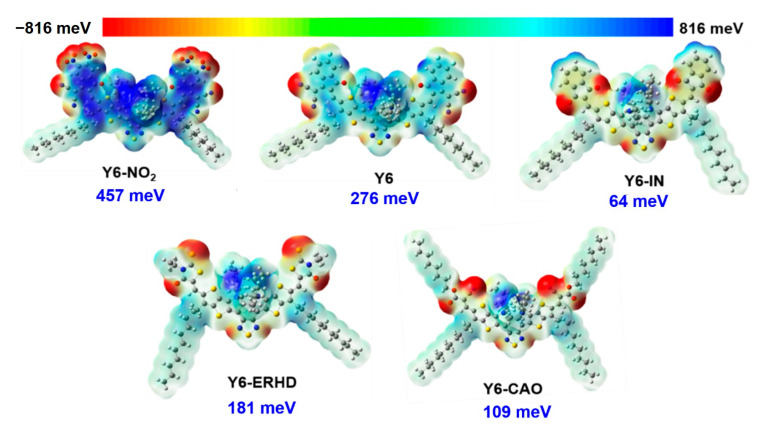
Averaged ESP maps of Y6-NO_2_, Y6, Y6-IN, Y6-ERHD, and Y6-CAO calculated at ωB97X/6-31+G(d)/PCM (ε = 3.0) level. The potential values are set from −816 (the deepest red) to 816 meV (the deepest blue).

Furthermore, the electrostatic potential (ESP) maps of Y6-NO_2_, Y6, Y6-IN, Y6-ERHD, and Y6-CAO monomers are presented in Figure 2. Owing to the A_1_–DA_2_D–A_1_ type of molecular frame, the negatively charged regions of these molecules are mainly located at the terminal group and the central benzothiadiazole (BT) unit, while the thiophene rings and side alkyl chains on the skeleton are positive. From Y6-IN to Y6 to Y6-NO_2_, with the increasing of the electron-withdrawing ability of the terminal groups, the averaged ESP of them increases from 64 meV to 457 meV. Because of the significant differences of the structures of terminal groups of Y6-ERHD and Y6-CAO (changed conjugated skeleton), both the averaged ESPs of two molecules are smaller than that of Y6. Generally, the electron acceptance ability is worse with smaller averaged ESP, so Y6-IN, Y6-ERHD, and Y6-CAO are not better acceptors from this perspective. 

### 2.2. FMO and Gap Energy

**Figure 3:**
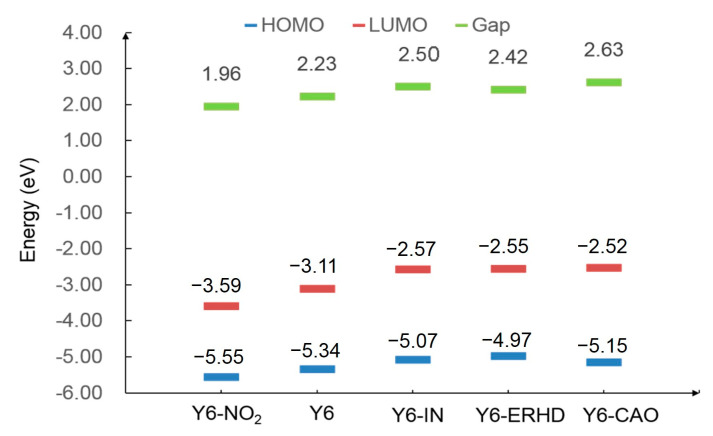
Calculated LUMO, HOMO, and gap energy of Y6-NO_2_, Y6, Y6-IN, Y6-ERHD, and Y6-CAO, obtained at ωB97X/6-31+G(d)/PCM theory level.

The HOMO, LUMO, and gap energy are shown in Figure 3. As the electron-withdrawing ability of the terminal acceptor unit decreases, the energy gaps of these acceptors generally becomes higher (except for Y6-ERHD) and the energy of the LUMO increases. This may lead to the rise of V_OC_. Y6-NO_2_ has the smallest energy gap and the lowest LUMO due to the strongest electron-withdrawing ability of -NO_2_. In addition, the HOMO energy generally decreases towards the stronger electron-withdrawing ability of the terminal units. This may not be favorable for hole transfer.

### 2.3. UV-Vis Spectra

**Figure 4:**
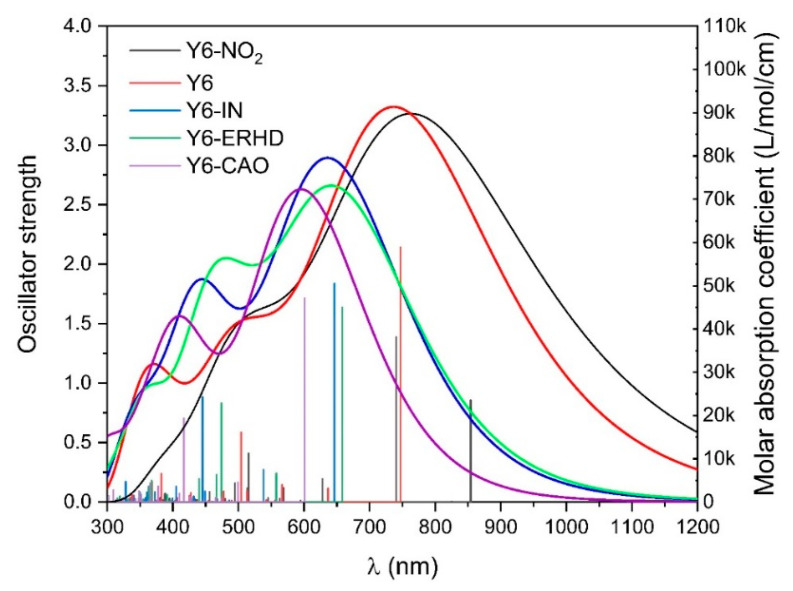
UV-Vis spectra of Y6-NO_2_, Y6, Y6-IN, Y6-ERHD and Y6-CAO, based on ωB97X/6-31+G(d)/PCM.

**Figure 5:**
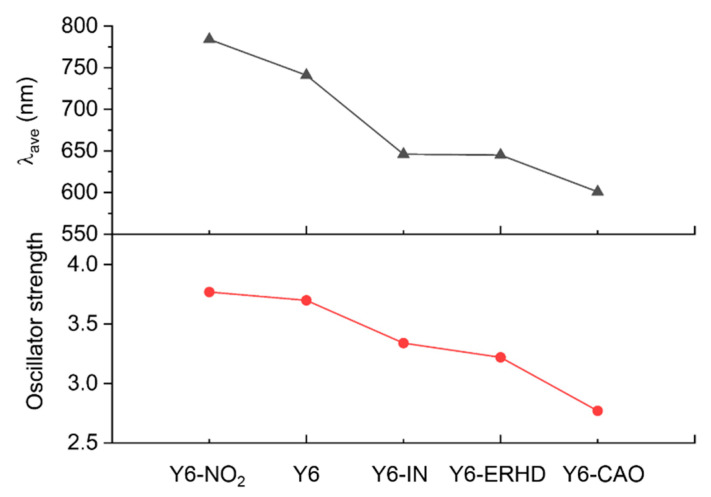
Wavelength of maximum absorption peaks and the total oscillator strength of five molecules in the visible and near-infrared regions.

As can be seen from Figure 4, the simulated UV-Vis spectra of Y6-NO_2_, Y6, Y6-IN, Y6-ERHD, and Y6-CAO in film are provided. Table S1 shows the results of the calculated excited states of these acceptors. For Y6-NO_2_, Y6, Y6-IN, Y6-ERHD, and Y6-CAO, the wavelength of the maximum absorption peaks is at 784.17, 741.48, 646.09, 645.41, and 601.25 nm, respectively, and the total oscillator strength in the visible region is 3.77, 3.70, 3.34, 3.22, and 2.77, correspondingly. Compared with Y6, the wavelength of the absorption peak of Y6-NO_2_ is red-shifted by 43 nm and its total oscillator strength is increased by 0.07. With the rise of electron-withdrawing ability (Y6-CAO < Y6-ERHD < Y6-IN < Y6 < Y6-NO_2_), the oscillator strength increases and the UV-Vis spectrum is red-shifted (see Figure 4 and Figure 5), which is consistent with the reduction of the energy gaps. The wavelength of absorption peaks and oscillator strength of Y6-NO_2_ are larger than those of Y6, owing to the strongest electron-withdrawing ability. 

### 2.4. Exciton Binding Energy

**Figure 6:**
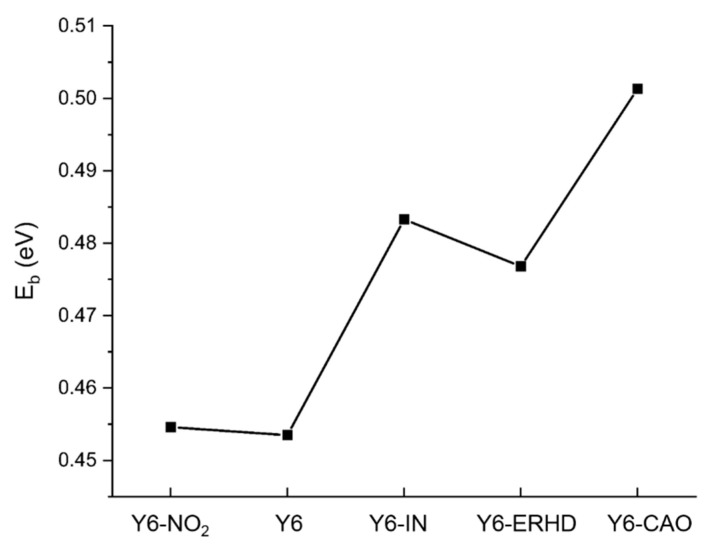
Calculated exciton binding energy of Y6-NO_2_, Y6, Y6-IN, Y6-ERHD, and Y6-CAO, obtained with ωB97X/6-31+G(d) in film (ε=3.0).

The exciton binding energy is the energy cost to separate excitons into free holes and electrons in organic solar cells. The small exciton binding energy should be beneficial to charge separation, Jsc, and PCE [20]. We provide the calculated exciton binding energy of Y6-NO_2_, Y6, Y6-IN, Y6-ERHD, and Y6-CAO, obtained with ωB97X/6-31+G(d) in film, as shown in Figure 6. The exciton binding energy is calculated with Equations (5)–(7). The values of Y6-NO_2_, Y6, Y6-IN, Y6-ERHD and Y6-CAO are 0.455, 0.454, 0.483, 0.477, and 0.501 eV, respectively. Y6 possesses the smallest exciton binding energy, and the E_b_ of Y6-NO_2_ is similar to that of Y6. 

### 2.5. Reorganization Energy of Electron Transfer

**Figure 7:**
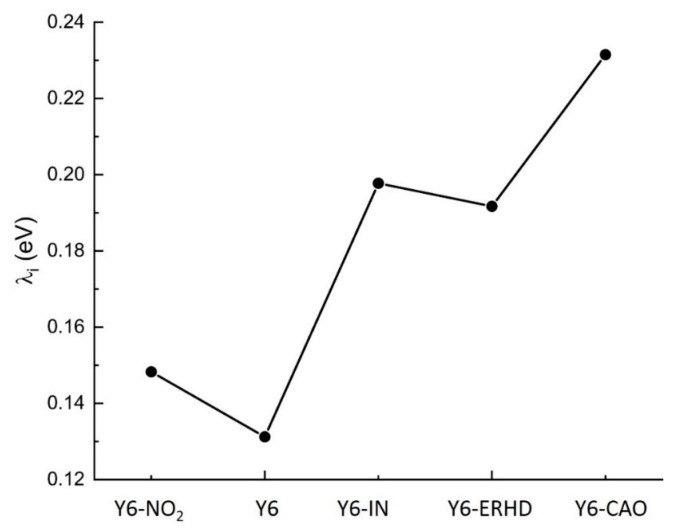
Calculated inner reorganization energies (eV) of Y6-NO_2_, Y6, Y6-IN, Y6-ERHD, and Y6-CAO by using B3LYP/6-31G(d) in film.

Reorganization energy (λ) is an important parameter influencing charge mobility, and it contains both inner and external parts. The inner reorganization energy (λ_i_) is the energy for the change of geometric structure in the process of charge transport. The inner reorganization energy of Y6-NO_2_, Y6, Y6-IN, Y6-ERHD, and Y6-CAO was calculated with Equation (8) by using B3LYP/6-31G(d), as displayed in Figure 7. The inner reorganization energy of Y6-NO_2_, Y6, Y6-IN, Y6-ERHD, and Y6-CAO is 0.15, 0.13, 0.20, 0.19, and 0.23 eV, respectively. Similar to the E_b_s of these molecules, Y6 and Y6-NO_2_ possess the smallest and second-smallest inner reorganization energy, correspondingly. 

The external reorganization energy (λ_o_) means the energy required for the nuclear relaxation and electron polarization of surrounding molecules during charge transfer. The results are calculated according to Equation (9), as shown in Table 2. Y6 and Y6-CAO have the smallest and biggest total reorganization energy, respectively. New molecules with modified terminal groups all have a bigger total reorganization energy than Y6. 

### 2.6. Electron/Hole Distribution of Monomers

**Figure 8:**
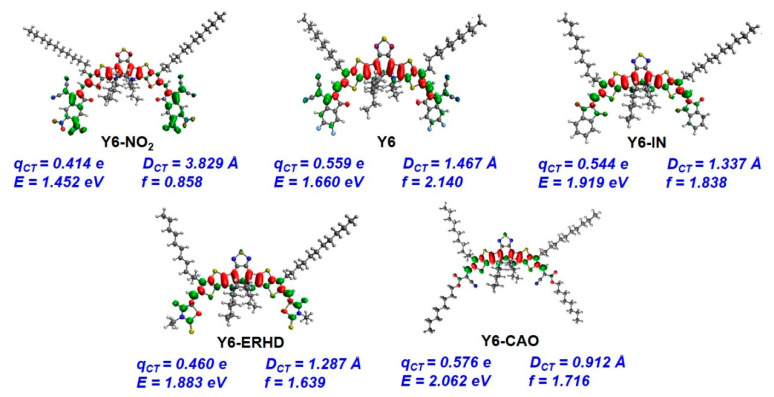
Electron/hole distributions of the first bright local excited (LE) state of Y6-NO_2_, Y6, Y6-IN, Y6-ERHD, and Y6-CAO. The intramolecular charge-transfer amount (*q_CT_*), intramolecular electron transition distance (*D_CT_*), oscillator strength (*f*), and local excited state energy (*E*) of five molecules are displayed. Green and red represent hole and electron, respectively.

The electron/hole distributions of the first LE states of five molecules are shown in Figure 8. The intramolecular charge-transfer amounts (*q_CT_*) of five molecules are different (0.414–0.576 e), indicating that changing the terminal acceptor unit affects *q_CT_.* The intramolecular charge transition distance (*D_CT_*) of Y6-NO_2_, Y6, Y6-IN, Y6-ERHD, and Y6-CAO is 3.83, 1.47, 1.34, 1.29, and 0.91 Å, respectively, demonstrating that *D_CT_* rises with the enhanced electron-withdrawing ability of terminal groups. So, the intermolecular charge transfer in Y6-NO_2_ may be easier than that in Y6. 

### 2.7. Quadrupole Moment along π-π Stacking Direction, ΔLUMO and ΔHOMO

**Figure 9:**
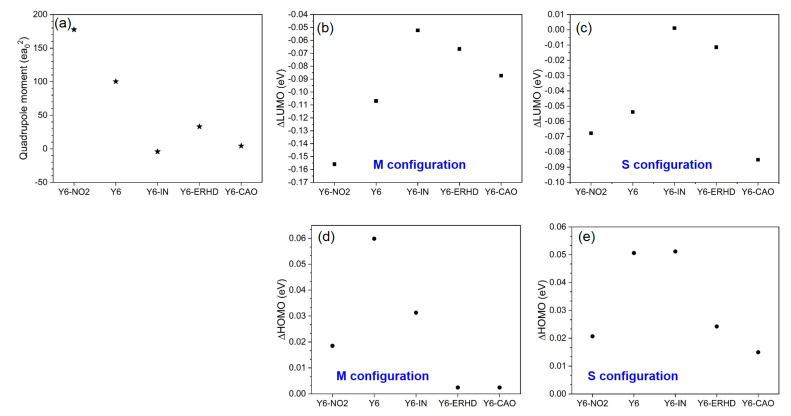
(**a**) Quadrupole moment along π-π stacking direction (Q_π_) of Y6-NO_2_, Y6, Y6-IN, Y6-ERHD and Y6-CAO calculated by Gaussian 09; (**b**) ΔLUMO between monomer and corresponding dimers with M configuration; (**c**) ΔLUMO between monomer and corresponding dimers with S configuration; (**d**) ΔHOMO between monomer and corresponding dimers with M configuration; (**e**) ΔHOMO between monomer and corresponding dimers with S configuration.

Y6-NO_2_, Y6 and Y6-ERHD and Y6-CAO have a positive Q_π,_ whereas Y6-IN has a negative Q_π_ as shown in Figure 9. Those of Y6-NO_2_ (177.55 ea_0_^2^), Y6 (100.30 ea_0_^2^) and Y6-ERHD (33.09 ea_0_^2^) are big. Except for Y6-CAO (4.36 ea_0_^2^), the ΔLUMO between monomers and corresponding dimers with M configurations is quite different from that between monomers and corresponding dimers with S configurations. Y6-NO_2_, Y6 and Y6-ERHD show a large positive Q_π,_ which can account for the large energetic shift with different molecular orientations, as observed in M and S configurations [21]. However, there is no law for ΔHOMO.

### 2.8. Binding Energy of Dimers and Intermolecular Distance of Charge Transfer

**Figure 10:**
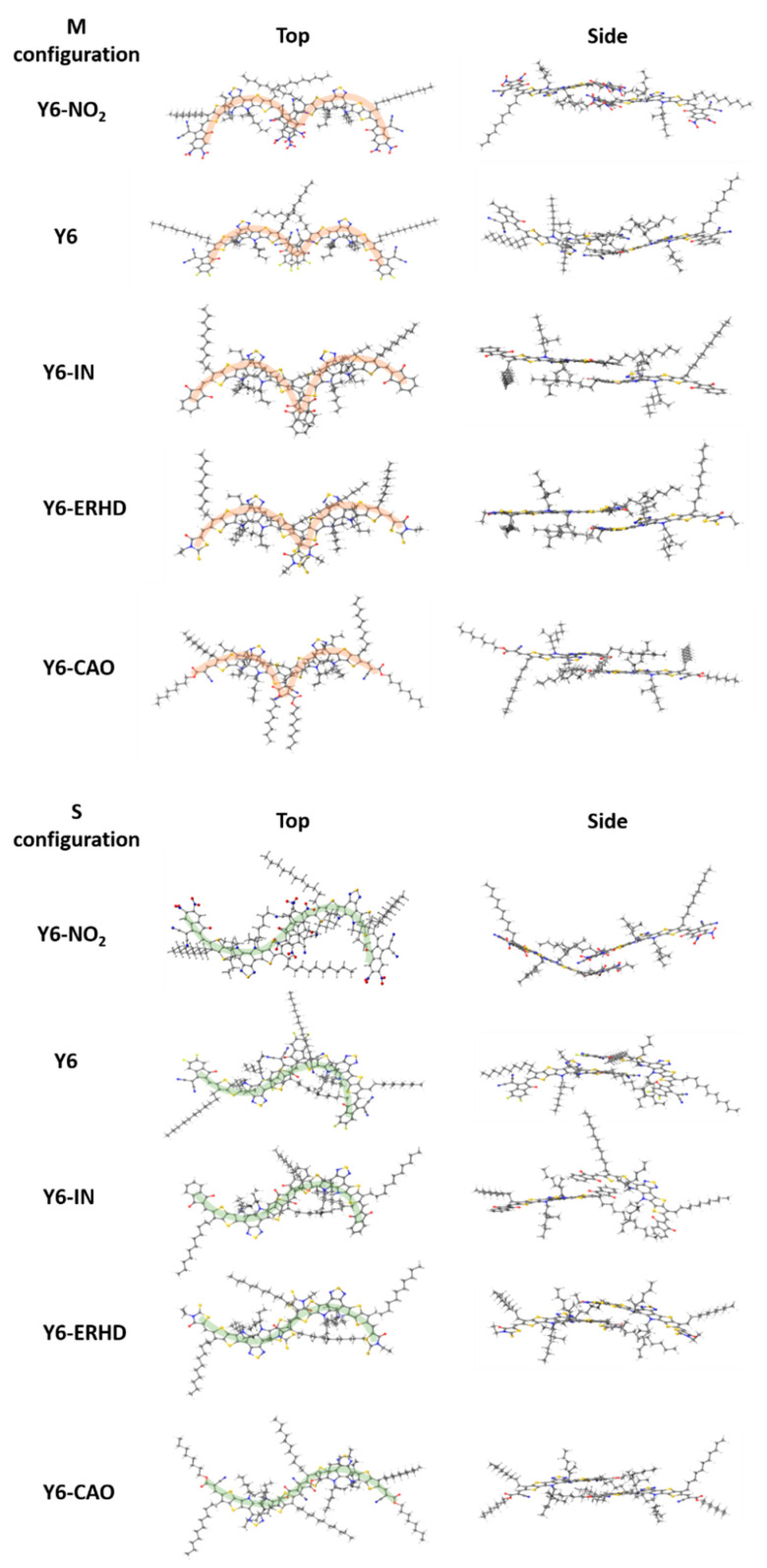
M- and S-configurations of Y6-NO_2_, Y6, Y6-IN, Y6-ERHD, and Y6-CAO dimers.

**Figure 11:**
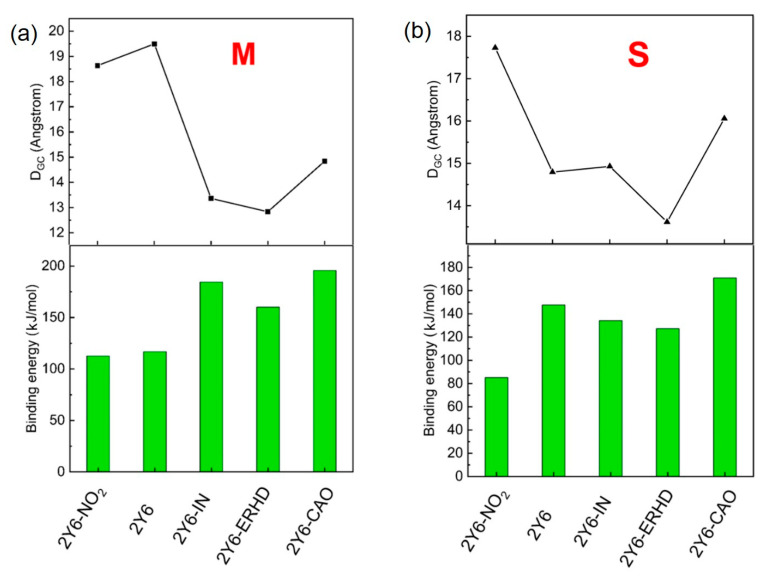
Binding energy and the geometry center distance (*D_GC_*) of Y6-NO_2_, Y6, Y6-IN, Y6-ERHD and Y6-CAO-based dimers: (**a**) M configuration; (**b**) S configuration.

**Figure 12:**
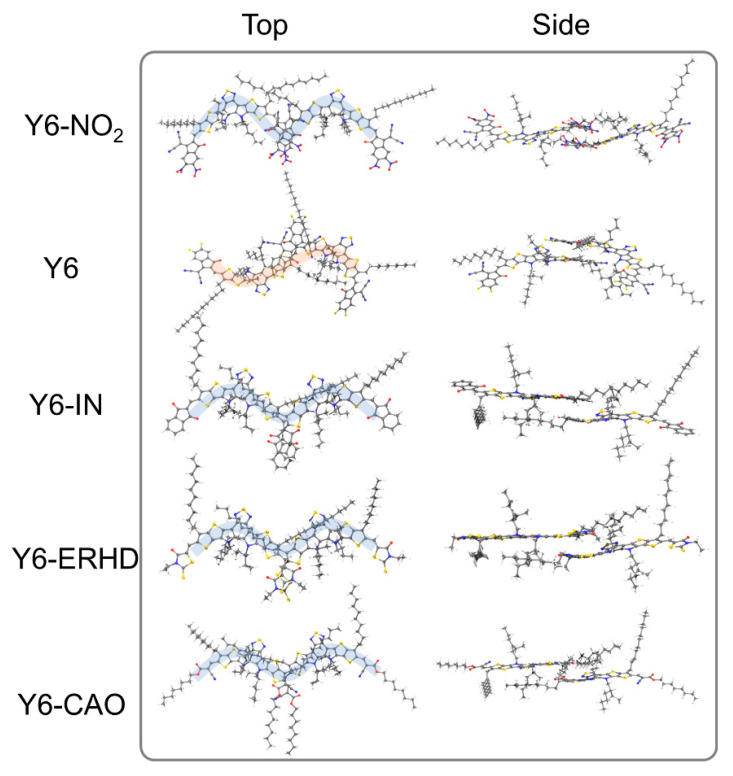
Stable configuration of Y6-NO_2_ (M), Y6 (S), Y6-IN (M), Y6-ERHD (M), and Y6-CAO (M) dimers, optimized at ωB97XD/6-31G(d)/PCM theory level.

In this section, the binding energies and the geometry center distances (*D_GC_*) of Y6-NO_2_, Y6, Y6-IN, Y6-ERHD, and Y6-CAO dimers with M and S configurations (see Figure 10) are presented, as shown in Figure 11 and Tables S2 and S3. We note that all the side alkyl chains are not contained when measuring the geometry center distances since they do not have noticeable contributions to the FMOs. Figure 11 shows that the Y6-IN and Y6-CAO dimers have larger binding energies, suggesting that the Y6-IN and Y6-CAO molecules may be more tightly packed in the dimer. In addition, Y6-NO_2_, Y6-IN, Y6-ERHD, and Y6-CAO M-configuration dimers have larger binding energy, showing that they may be more stable than S-configuration dimers, except for Y6. On the contrary, for Y6, the S-configuration dimer is more stable, and this is consistent with the experimental results [22]. Clearly, the change of the terminal group makes the stable configuration of the dimer transform from the S-type to the M-type. Figure 12 presents the stable dimer configurations of these molecules. We shall focus on these dimers for charge transfer and mobility. We also calculated the dihedral angles of the side chains of the dimers with both M and S configurations to understand the effect of side chains on molecular stacking in dimers (SI Figures S1 and S2). However, we could not find any law regarding the dihedral angles of side chains. Almost in all the dimers with both M and S configurations, the orientations of the side chains near the end group stacking of the dimer tend to be horizontal, and the orientations of the side chains far from the end group stacking are arbitrary [23].

### 2.9. Charge Carrier Mobility

The charge-transfer distance in these dimers, shown in Table 3, is estimated by using the geometry center distance. In general, a short charge-transfer distance is conducive for enhancing electronic coupling but may not be beneficial for carrier mobility (see Equation (13)). On the other hand, electronic coupling represents the overlap between the FMOs of adjacent molecules and is critical for charge carrier mobility. The one-dimensional charge carrier mobility is calculated by assuming that the charge carrier can only jump to its nearest neighbor and its continuous jumps are not correlated [24]. The charge-transfer rate constants, electronic coupling, charge carrier mobility, and other related parameters of stable dimers are displayed in Table 3. We also provide these data for other dimer configurations in SI Table S4. 

Table 3 demonstrates that except for Y6-ERHD, the charge-transfer rate constant and electronic coupling of Y6-NO_2_, Y6-IN, and Y6-CAO are larger than those of Y6. The electron mobility of Y6-NO_2_ (5.87 × 10^−1^ cm^2^ V^−1^ s^−1^), Y6-IN (3.84 × 10^−1^ cm^2^ V^−1^ s^−1^), and Y6-CAO (3.76 × 10^−1^ cm^2^ V^−1^ s^−1^) is about six, four, and four times faster than that of Y6 (1.03 × 10^−1^ cm^2^ V^−1^ s^−1^), respectively. In contrast, the electron mobility of Y6-ERHD (1.00 × 10^−3^ cm^2^ V^−1^ s^−1^) is only one percent of that of Y6 (1.03 × 10^−1^ cm^2^ V^−1^ s^−1^). 

**Figure 13:**
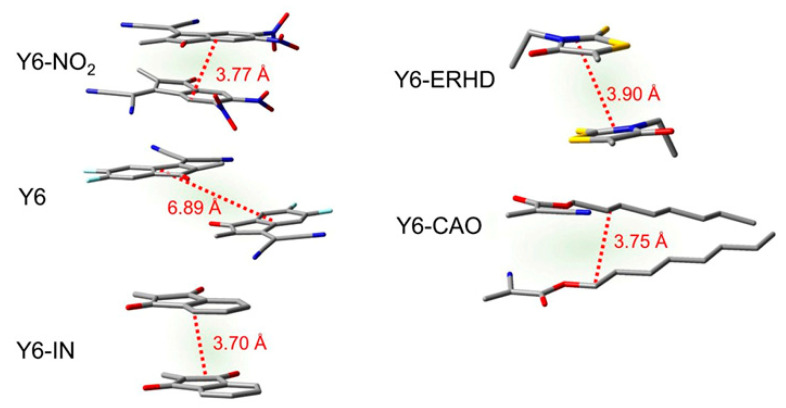
End-group stacking and geometric center distances of the dimer configurations.

To further understand the reason for the differences in electronic coupling and electron mobility among these dimers, we measured the geometric center distances between the end groups in these dimers, as shown in Figure 13. The end group geometric center distances of Y6-NO_2_ (3.77 Å) and Y6-IN (3.70 Å) are shorter than that of Y6 (6.89 Å), resulting in stronger electron coupling. On the other hand, although the geometric center distance of Y6-ERHD (3.90 Å) is much shorter than that of Y6 (6.89 Å), its terminal conjugated ring is too small when they stack with each other, leading to very low electronic coupling and a low charge-transfer rate constant. For Y6-CAO, the flexible end groups make the stacks less rigid, leading to high electronic coupling and electron mobility. Clearly, Y6-NO_2_ possesses the fastest electron mobility because it has strong electronic coupling and the longest charge-transfer distance simultaneously. Based on these results, we can conclude that the change of terminal group has a great influence on the electron mobility of these Y6 derivatives. 

## 3. Methods

All molecules were constructed with GaussView version 5.0 [25]. All calculations were calculated by using Guassian09 Rev E.01 software package [26]. In consideration of environmental effects in the thin film of OSCs, we adopted an integral equation formalism variant–polarizable continuum model (IEF-PCM), and the dielectric constant was set to 3.0 [27,28]. The Y6-NO_2_, Y6-IN, Y6-ERHD, and Y6-CAO molecules were initially optimized with the combination of the B3LYP [29] hybrid density functional and 6-31G(d) basis set, which has proven to be reliable for the geometric optimization of organic molecules [30,31]. This combination also was used for the calculation of the inner reorganization energy. If not mentioned otherwise, the 6-31+G(d) basis set was default for all other calculations since it was more accurate for excited state calculations and weak interactions [32]. We also selected the range separated hybrid (RSH) density functional ωB97X with tunable parameter ω [33] because ωB97X has proven to be accurate for Y6 and its derivatives [34]. By tuning the parameter ω with IEF-PCM, the gained absolute values of HOMO and LUMO energies are almost equal to those of EA and IP. We used the following equations to optimize ω [33]:

(1) $$J^{2}\left(\omega \right)=J_{N}^{2}\left(\omega \right)+J_{N+1}^{2}\left(\omega \right)$$

(2) $$J_{N}^{2}\left(\omega \right)={\left[\varepsilon_{HOMO}^{\omega }\left(N\right)+E^{\omega }\left(N-1\right)-E^{\omega }\left(N\right)\right]}^{2}$$

(3) $$J_{N+1}^{2}\left(\omega \right)={\left[\varepsilon_{HOMO}^{\omega }\left(N+1\right)+E^{\omega }\left(N\right)-E^{\omega }\left(N+1\right)\right]}^{2}$$

where $E^{\omega }\left(N\right)$ is the energy of the neutral system; $\varepsilon_{HOMO}^{\omega }\left(N\right)$ is the energy of the HOMO of the neutral system; $E^{\omega }\left(N+1\right)$ is the energy of the anion; and $E^{\omega }\left(N-1\right)$ is the energy of the cation. The optimized ω of Y6, Y6-NO_2_, Y6-IN, Y6-ERHD and Y6-CAO is 0.016, 0.013, 0.015, 0.014, and 0.016, respectively.

Multiwfn 3.8 software was adopted to simulate absorption spectra with the output of TDDFT calculations [35]. The full width at half maximum (FWHM) of absorption peaks was default (0.6667 eV). For the wavelength of simulated absorption peaks, the following formula was used:

(4) $$\lambda_{ave}=\frac{\sum \lambda_{n}\times f_{n}}{\sum f_{n}}$$

where $\lambda_{ave}$ represents the wavelength of the absorption peak; $f_{n}$ means the oscillator strength of the *n*th excited state; and $\lambda_{n}$ donates the wavelength of the *n*th excited state [34]. 

Exciton binding energy ($E_{b}$) in thin films is a key factor determining charge transfer and separation. We used the formulas as follows [36]:

(5) $$E_{b}=E_{g}^{\mathrm{fund}}-E_{g}^{\mathrm{opt}}$$

(6) $$E_{g}^{\mathrm{fund}}=E\left(N-1\right)+E\left(N+1\right)-2E\left(N\right)$$

(7) $$E_{g}^{\mathrm{opt}}=E\left(N,\mathrm{Excited}\right)-E\left(N\right)\;$$

where $E_{b}$ donates exciton binding energy; $E_{g}^{\mathrm{opt}}$ means the energy of the optical gap; $E_{g}^{\mathrm{fund}}$ is the energy of the fundamental gap; N represents a neutral system; N + 1 is a negative; and N − 1 is a positive.

The inner reorganization energy is the energy required for the change of nuclear geometric structure during charge transfer. The following equation was used to calculate the inner reorganization energy [37,38]:

(8) $$\lambda_{i,\;e}=\left[E^{0}\left(A^{-}\right)-E^{0}\left(A^{0}\right)\right]+\left[E^{-}\left(A^{0}\right)-E^{-}\left(A^{-}\right)\right]$$

where $\lambda_{i,\;e}$ means the inner reorganization energy during electron transfer; $E^{0}\left(A^{-}\right)$ is the total energy of the neutral acceptor in the optimized geometry of the anion; $E^{0}\left(A^{0}\right)$ represents the total energy of the neutral acceptor in the optimized geometry of the neutral molecule; $E^{-}\left(A^{0}\right)$ denotes the energy of the negative acceptor in the optimized geometry of the neutral molecule; and $E^{-}\left(A^{-}\right)$ is energy of the negative acceptor in the optimized geometry of the anion. 

Taking into account environmental impact, external reorganization energy (λ_o_) is defined as the energy required for the molecular nuclear relaxation and electronic polarization of the surrounding molecules of acceptor dimers during the charge-transfer process. The formula for calculating the external reorganization energy (λ_o_) is [39,40] is:

(9) $$\lambda_{o}\;=\frac{{\left(\Delta q\right)}^{2}}{8\pi \varepsilon_{0}}(\frac{1}{\varepsilon_{opt}}-\frac{1}{\varepsilon_{s}})(\frac{1}{R_{D}}+\frac{1}{R_{A}}-\frac{2}{r_{DA}})$$

where *Δq* is the transferred element charge; *ε*_0_ is the vacuum permittivity (*ε*_0_ = 8.85 × 10^−12^ F/m); and *ε_opt_* and *ε_s_* represent the optical and static permittivity of the solvents, respectively, which were set to 2.0 and 3.0, correspondingly. In addition, *R_D_* and *R_A_* donate the effective radius of the donor and acceptor, respectively. In these dimers, *R_D_* is equal to *R_A_*, and *r_DA_* was set to the effective radius of the dimers. 

Basis set superposition error (BSSE) correction was applied to correct the binding energy of dimers. The binding energy was obtained by the following equation [41]:

(10) $$E_{binding}=E_{AB}-E_{A}-E_{B}+E_{BSSE}$$

where $E_{AB}$ is the total energy of the dimer; $E_{A}$ represents the energy of monomer *A*; $E_{B}$ denotes the energy of monomer *B*; and $E_{BSSE}$ means the BSSE energy. 

The semi-classical Marcus formula is written as follows [39,40]:

(11) $$k=\frac{2\pi }{\hbar }V^{2}\left(\frac{1}{\sqrt{4\pi \lambda k_{B}T}}\right)\exp\left(-\frac{\lambda }{4k_{B}T}\right)\;$$

where $k_{B}$ is the Boltzmann constant; V denotes the electronic coupling, T represents the absolute temperature and is set to 298.15 K; λ means the reorganization energy, and in this paper, it is the sum of the inner reorganization energy and external reorganization energy; ℏ is the reduced Planck constant; and the free energy difference between initial and final states was set to zero.

The electronic coupling was defined by the following equation [42]:

(12) $$V=\frac{H_{if}-S_{if}\left(H_{ii}+H_{ff}\right)/2}{1-S_{if}^{2}}$$

where $H_{if}$ means the non-diagonal element of the two-state Hamiltonian matrix; $H_{ii}$/$H_{ff}$ denotes the Hamiltonian of the initial/final state of charge transfer, respectively; and $S_{if}$ is the overlap between initial and final states. The overall electronic coupling was calculated at CAM-B3LYP/6-31G(d) level of theory [43]. 

The carrier mobility could be computed by the Einstein–Smoluchowski formula [44]:

(13) $$\mu =\frac{eD}{k_{B}T}\;$$

where e is the elementary charge; D means the diffusion coefficient of the charge carrier; for the one-dimensional case,$\;D=r^{2}k/2$ (supposing that the charge carrier can only jump to its nearest neighbor sites and its continuous jumps are uncorrelated) [24]; k denotes the charge-transfer rate constant; and r is the charge-transfer distance between two adjacent molecules, which is estimated by the distance between geometry centers of neighboring molecules.

In order to simulate molecular stacking, we modeled the dimer configuration of these designed molecules, which is simple but very useful. Moreover, we assumed that the charge carrier can only jump to the nearest location, which is reasonable [45]. So, only dimer configurations that contain two neighboring monomers were considered in this work. There are different configurations for dimers, but experimental results have shown that the terminal-to-terminal stacking of the dimers of Y6 and its derivatives are dominant [43]. Therefore, only the dimers with M- and S-shaped configurations of these five molecules, as shown in Figure 10, have been optimized. 

## 4. Conclusions

Using DFT and TD-DFT calculations, we have investigated the effects of the changes of the terminal groups of Y6 on physical and optical properties. The dipole moments, averaged ESPs, absorption spectra in the visible and near-infrared regions, and reorganization energy of these molecules change greatly due to the variations in terminal groups. As electron-withdrawing ability of terminal group increases, the value of gap of them becomes lower. Therefore, the wavelengths of the main absorption peaks of UV-Vis spectra red-shifts and the total oscillator strength rise. Simultaneously, the LUMO energy also reduces, which may lead to the decrease in Voc. Last but not least, the electron mobility of Y6-NO_2_, Y6-IN, and Y6-CAO is enhanced to be about six, four, and four times than that of Y6, respectively. Overall, considering all computed results, Y6-NO_2_ seems to be a promising NFA because of its exciton binding energy, longer intramolecular charge-transfer distance, stronger dipole moment, higher averaged ESP, enhanced spectrum, and faster electron mobility. This study can provide guidance for future theoretical and experimental research on the modification of Y6.

## Figures and Tables

**Table 1:** Calculated dipole moments of Y6-NO_2_, Y6, Y6-IN, Y6-ERHD, and Y6-CAO gained with B3LYP/6-31G(d)/PCM. Unit: Debye.

Y6-NO_2_	Y6	Y6-IN	Y6-ERHD	Y6-CAO
15.56	0.82	4.16	6.31	2.96

**Table 2:** Reorganization energies of Y6-NO_2_, Y6, Y6-IN, Y6-ERHD, and Y6-CAO, obtained with the B3LYP/6-31G(d)/PCM (*ε* = 3.0) theory level. Unit: eV.

System	λ_i_	λ_o_	λ
Y6-NO_2_	0.15	0.19	0.34
Y6	0.13	0.16	0.29
Y6-IN	0.20	0.15	0.35
Y6-ERHD	0.19	0.14	0.33
Y6-CAO	0.23	0.15	0.38

**Table 3:** Charge-transfer distance (r, in Å), absolute value of electronic coupling (|V_e_| in meV), charge-transfer rate constant (k_e_, in s^−1^), reorganization energy (λ, in eV) of electron transfer, charge carrier mobility (μ_e_, in cm^2^ V^−1^ s^−1^) of the stable dimer of Y6-NO_2_, Y6, Y6-IN, Y6-ERHD and Y6-CAO.

	r	|V_e_|	λ	k_e_	μ_e_
Y6-NO_2_-M-dimer	18.63	27.89	0.33	8.69 × 10^11^	5.87 × 10^−1^
Y6-S-dimer	14.79	11.43	0.29	2.42 × 10^11^	1.03 × 10^−1^
Y6-IN-M-dimer	13.37	33.75	0.35	1.11 × 10^12^	3.84 × 10^−1^
Y6-ERHD-M-dimer	12.84	1.57	0.33	2.83 × 10^9^	1.00 × 10^−3^
Y6-CAO-M-dimer	14.84	37.00	0.38	8.77 × 10^11^	3.76 × 10^−1^

## Data Availability

Not applicable.

## Supplementary Materials

The following supporting information can be downloaded at: https://www.mdpi.com/article/10.3390/ijms24108610/s1.

## References

[1] Cui Y., Xu Y., Yao H., Bi P., Hong L., Zhang J., Zu Y., Zhang T., Qin J., Ren J. (2021). Single-Junction Organic Photovoltaic Cell with 19% Efficiency. Adv. Mater..

[2] Yuan J., Zhang Y., Zhou L., Zhang G., Yip H.-L., Lau T.-K., Lu X., Zhu C., Peng H., Johnson P.A. (2019). Single-Junction Organic Solar Cell with over 15% Efficiency Using Fused-Ring Acceptor with Electron-Deficient Core. Joule.

[3] Hou J., Inganas O., Friend R.H., Gao F. (2018). Organic solar cells based on non-fullerene acceptors. Nat. Mater..

[4] Fei Z., Eisner F.D., Jiao X., Azzouzi M., Rohr J.A., Han Y., Shahid M., Chesman A.S.R., Easton C.D., McNeill C.R. (2018). An Alkylated Indacenodithieno[3,2-b]thiophene-Based Nonfullerene Acceptor with High Crystallinity Exhibiting Single Junction Solar Cell Efficiencies Greater than 13% with Low Voltage Losses. Adv. Mater..

[5] Hong L., Yao H., Wu Z., Cui Y., Zhang T., Xu Y., Yu R., Liao Q., Gao B., Xian K. (2019). Eco-Compatible Solvent-Processed Organic Photovoltaic Cells with Over 16% Efficiency. Adv. Mater..

[6] Li C., Zhou J., Song J., Xu J., Zhang H., Zhang X., Guo J., Zhu L., Wei D., Han G. (2021). Non-fullerene acceptors with branched side chains and improved molecular packing to exceed 18% efficiency in organic solar cells. Nat. Energy.

[7] Gao W., Fu H., Li Y., Lin F., Sun R., Wu Z., Wu X., Zhong C., Min J., Luo J. (2021). Asymmetric Acceptors Enabling Organic Solar Cells to Achieve an over 17% Efficiency: Conformation Effects on Regulating Molecular Properties and Suppressing Nonradiative Energy Loss. Adv. Energy Mater..

[8] Wang R., Yuan J., Wang R., Han G., Huang T., Huang W., Xue J., Wang H.C., Zhang C., Zhu C. (2019). Rational Tuning of Molecular Interaction and Energy Level Alignment Enables High-Performance Organic Photovoltaics. Adv. Mater..

[9] Zhang Z., Li Y., Cai G., Zhang Y., Lu X., Lin Y. (2020). Selenium Heterocyclic Electron Acceptor with Small Urbach Energy for As-Cast High-Performance Organic Solar Cells. J. Am. Chem. Soc..

[10] Cui Y., Yao H., Zhang J., Zhang T., Wang Y., Hong L., Xian K., Xu B., Zhang S., Peng J. (2019). Over 16% efficiency organic photovoltaic cells enabled by a chlorinated acceptor with increased open-circuit voltages. Nat. Commun..

[11] Hu D., Yang Q., Zheng Y., Tang H., Chung S., Singh R., Lv J., Fu J., Kan Z., Qin B. (2021). 15.3% Efficiency All-Small-Molecule Organic Solar Cells Achieved by a Locally Asymmetric F, Cl Disubstitution Strategy. Adv. Sci..

[12] Lu H., Jin H., Huang H., Liu W., Tang Z., Zhang J., Bo Z. (2021). High-Efficiency Organic Solar Cells Based on Asymmetric Acceptors Bearing One 3D Shape-Persistent Terminal Group. Adv. Funct. Mater..

[13] Li S., Zhan L., Yao N., Xia X., Chen Z., Yang W., He C., Zuo L., Shi M., Zhu H. (2021). Unveiling structure-performance relationships from multi-scales in non-fullerene organic photovoltaics. Nat. Commun..

[14] Cao C., Lai H., Chen H., Zhu Y., Pu M., Zheng N., He F. (2021). Over 17.5% efficiency ternary organic solar cells with enhanced photon utilization via a medium band gap non-fullerene acceptor. J. Mater. Chem. A.

[15] Jones R.O., Gunnarsson O. (1989). The density functional formalism, its applications and prospects. Rev. Mod. Phys..

[16] Runge E., Gross E.K.U. (1984). Density functional theory for time-dependent systems. Phys. Rev. Lett..

[17] Chai J.D., Head-Gordon M. (2008). Systematic optimization of long-range corrected hybrid density functionals. J. Chem. Phys..

[18] Xu B., Yi X., Huang T.Y., Zheng Z., Zhang J., Salehi A., Coropceanu V., Ho C.H.Y., Marder S.R., Toney M.F. (2018). Donor Conjugated Polymers with Polar Side Chain Groups: The Role of Dielectric Constant and Energetic Disorder on Photovoltaic Performance. Adv. Funct. Mater..

[19] Takacs C.J., Sun Y., Welch G.C., Perez L.A., Liu X., Wen W., Bazan G.C., Heeger A.J. (2012). Solar cell efficiency, self-assembly, and dipole-dipole interactions of isomorphic narrow-band-gap molecules. J. Am. Chem. Soc..

[20] Kraner S., Prampolini G., Cuniberti G. (2017). Exciton Binding Energy in Molecular Triads. J. Phys. Chem. C.

[21] Fu Y., Lee T.H., Chin Y.C., Pacalaj R.A., Labanti C., Park S.Y., Dong Y., Cho H.W., Kim J.Y., Minami D. (2023). Molecular orientation-dependent energetic shifts in solution-processed non-fullerene acceptors and their impact on organic photovoltaic performance. Nat. Commun..

[22] Zhu W., Spencer A.P., Mukherjee S., Alzola J.M., Sangwan V.K., Amsterdam S.H., Swick S.M., Jones L.O., Heiber M.C., Herzing A.A. (2020). Crystallography, Morphology, Electronic Structure, and Transport in Non-Fullerene/Non-Indacenodithienothiophene Polymer:Y6 Solar Cells. J. Am. Chem. Soc..

[23] Zhong S., Yap B.K., Zhong Z., Ying L. (2022). Review on Y6-Based Semiconductor Materials and Their Future Development via Machine Learning. Crystals.

[24] Cornil J., Lemaur V., Calbert J.P., Brédas J.L. (2002). Charge Transport in Discotic Liquid Crystals: A Molecular Scale Description. Adv. Mater..

[25] Dennington R.K.T., Millam J. (2009). GaussView, Version 5.

[26] Frisch M.J., Trucks G.W., Schlegel H.B., Scuseria G.E., Robb M.A., Cheeseman J.R., Scalmani G., Barone V., Mennucci B., Petersson G.A. (2009). Gaussian 2009 Revision D.01.

[27] Jacobson L.D., Herbert J.M. (2010). Polarization-Bound Quasi-Continuum States Are Responsible for the “Blue Tail” in the Optical Absorption Spectrum of the Aqueous Electron. J. Am. Chem. Soc..

[28] Zheng S., Geva E., Dunietz B.D. (2013). Solvated Charge Transfer States of Functionalized Anthracene and Tetracyanoethylene Dimers: A Computational Study Based on a Range Separated Hybrid Functional and Charge Constrained Self-Consistent Field with Switching Gaussian Polarized Continuum Models. J. Chem. Theory Comput..

[29] Stephens P.J., Devlin F.J., Chabalowski C.F., Frisch M.J. (1994). Ab-Initio Calculation of Vibrational Absorption and Circular-Dichroism Spectra Using Density-Functional Force-Fields. J. Phys. Chem..

[30] Hehre W.J., Ditchfield R., Pople J.A. (1972). Self-Consistent Molecular-Orbital Methods.XII. Further Extensions of Gaussian-Type Basis Sets for Use in Molecular-Orbital Studies of Organic-Molecules. J. Chem. Phys..

[31] Al-shamiri H.A.S., Melhi S., Alosaimi E.H., El-Gammal B., Elhouichet H., Sakr M.A.S., Abou Kana M.T.H., Kandel H.M. (2023). Experimental and theoretical study of optical properties of pyrromethene (PM-597) laser dye in binary eco-friendly solvent. J. Phys. Org. Chem..

[32] McCarthy M., Lee K.L.K. (2020). Molecule Identification with Rotational Spectroscopy and Probabilistic Deep Learning. J. Phys. Chem. A.

[33] Sun H.T., Zhong C., Sun Z.R. (2016). Recent Advances in the Optimally “Tuned” Range-Separated Density Functional Theory. Acta Phys.-Chim. Sin..

[34] Qiu W., Zheng S. (2021). Designing and Screening High-Performance Non-Fullerene Acceptors: A Theoretical Exploration of Modified Y6. Solar Rrl.

[35] Shukla S., Srivastava A., Srivastava K., Tandon P., Jamalis J., Singh R.B. (2020). Non-covalent interactions and spectroscopic study of chalcone derivative 1-(4-chlorophenyl)-3-(5-methylfuran-2-yl) prop-2-en-1-one. J. Mol. Struct..

[36] Lee J.C., Chai J.D., Lin S.T. (2015). Assessment of density functional methods for exciton binding energies and related optoelectronic properties. RSC Adv..

[37] Nelsen S.F., Blackstock S.C., Kim Y. (1987). Estimation of Inner Shell Marcus Terms for Amino Nitrogen Compounds by Molecular Orbital Calculations. J. Am. Chem. Soc..

[38] Sammut D., Bugeja N., Szacilowski K., Magri D.C. (2022). Molecular engineering of fluorescent bichromophore 1,3,5-triaryl-Delta(2)-pyrazoline and 4-amino-1,8-naphthalimide molecular logic gates. New J. Chem..

[39] Marcus R.A. (1993). Electron transfer reactions in chemistry. Theory and experiment. Rev. Mod. Phys..

[40] Marcus R.A., Sutin N. (1985). Electron transfer in chemistry and biology. Biochim. Biophys. Acta.

[41] Komornicki A., Dixon D.A. (1992). Accurate proton affinities:Abinitioproton binding energies for N_2_, CO, CO_2_, and CH_4_. J. Chem. Phys..

[42] Valeev E.F., Coropceanu V., da Silva Filho D.A., Salman S., Brédas J.L. (2006). Effect of Electronic Polarization on Charge-Transport Parameters in Molecular Organic Semiconductors. J. Am. Chem. Soc..

[43] Mkoma S.L., Msambwa Y., Jacob F.R., Kiruri L.W., Kinunda G.A., Mlowe S., Deogratias G. (2022). Optical and electronic properties of para-functionalized triphenylamine-based dyes: A theoretical study. Struct. Chem..

[44] Coropceanu V., Cornil J., da Silva Filho D.A., Olivier Y., Silbey R., Bredas J.L. (2007). Charge Transport in Organic Semiconductors. Chem. Rev..

[45] An B., Wen K., Feng S., Pan X., Wu W., Guo X., Zhang J. (2018). Theoretical insights into the 1D-charge transport properties in a series of hexaazatrinaphthylene-based discotic molecules. J. Comput. Chem..

