# Supplementary material for: Increasing Charge Carrier Mobility through Modifications of Terminal Groups of Y6: A Theoretical Study
Source: Int J Mol Sci. 2023 May 11;24(10):8610. doi: 10.3390/ijms24108610 (PMC10218651; doi:10.3390/ijms24108610)
Supplement: Supplementary file 1 [file ijms-24-08610-s001.zip › ijms-2347941-supplementary.pdf]

SI:

Increasing charge carrier mobility through modifications of terminal groups of Y6: a theoretical study

Yunjie Xiang*, Chunlin Xu and Shaohui Zheng*

School of Materials and Energy

Chongqing Key Laboratory for Advanced Materials and Technologies of Clean Energies

Southwest University, Chongqing, China

Corresponding author: hao1986kx88@swu.edu.cn; shaohuizheng@swu.edu.cn

Table S1. Calculated excited states and oscillator strength of Y6-NO_2, Y6, Y6-IN, Y6-ERHD and Y6-CAO in film by using ωB97X functional.

	Excited State	Energy (eV)	λ(nm)	Oscillator strength
Y6-NO_2	1	1.4519	853.93	0.8588
	2	1.5023	825.32	0.0073
	3	1.6737	740.79	1.3879
	λ_{ave}	1.5813	784.17	2.2540
Y6	1	1.6595	747.1	2.1413
	2	1.9479	636.5	0.1146
	λ_{ave}	1.6723	741.48	2.2559
Y6-IN	1	1.9190	646.09	1.8378
	λ_{ave}	1.9190	646.09	1.8378
Y6-ERHD	1	1.8833	658.32	1.6356
	2	2.223	557.73	0.2408
	λ_{ave}	1.9213	645.41	1.8764
Y6-CAO	1	2.0621	601.25	1.7158
	λ_{ave}	2.0621	601.25	1.7158

Table S2. Binding energies and the geometry center distances (D_{GC}) of Y6-NO_2, Y6, Y6-IN, Y6-ERHD and Y6-CAO based dimers with M-configuration obtained by WB97XD with 6-31G (d) basis set in dibutylether (Ɛ=3.0). Unit: kJ/mol for binding energy; Å for D_{GC}.

System	Binding energy	D_{GC}
2Y6-NO_2	112.62	18.63
2Y6	116.87	19.49
2 Y6-IN	184.57	13.37
2Y6-ERHD	160.14	12.84
2Y6-CAO	195.73	14.84

Table S3. Binding energies and the geometry center distances (D_{GC}) of Y6-NO_2, Y6, Y6-IN, Y6-ERHD and Y6-CAO based dimers with S-configuration obtained by WB97XD with 6-31G (d) basis set in dibutylether (Ɛ=3.0). Unit: kJ/mol for binding energy; Å for D_{GC}.

System	Binding energy	D_{GC}
2Y6-NO2	85.19	17.73
2Y6	147.65	14.80
2 Y6-IN	134.18	14.93
2Y6-ERHD	127.32	13.62
2Y6-CAO	170.94	16.06

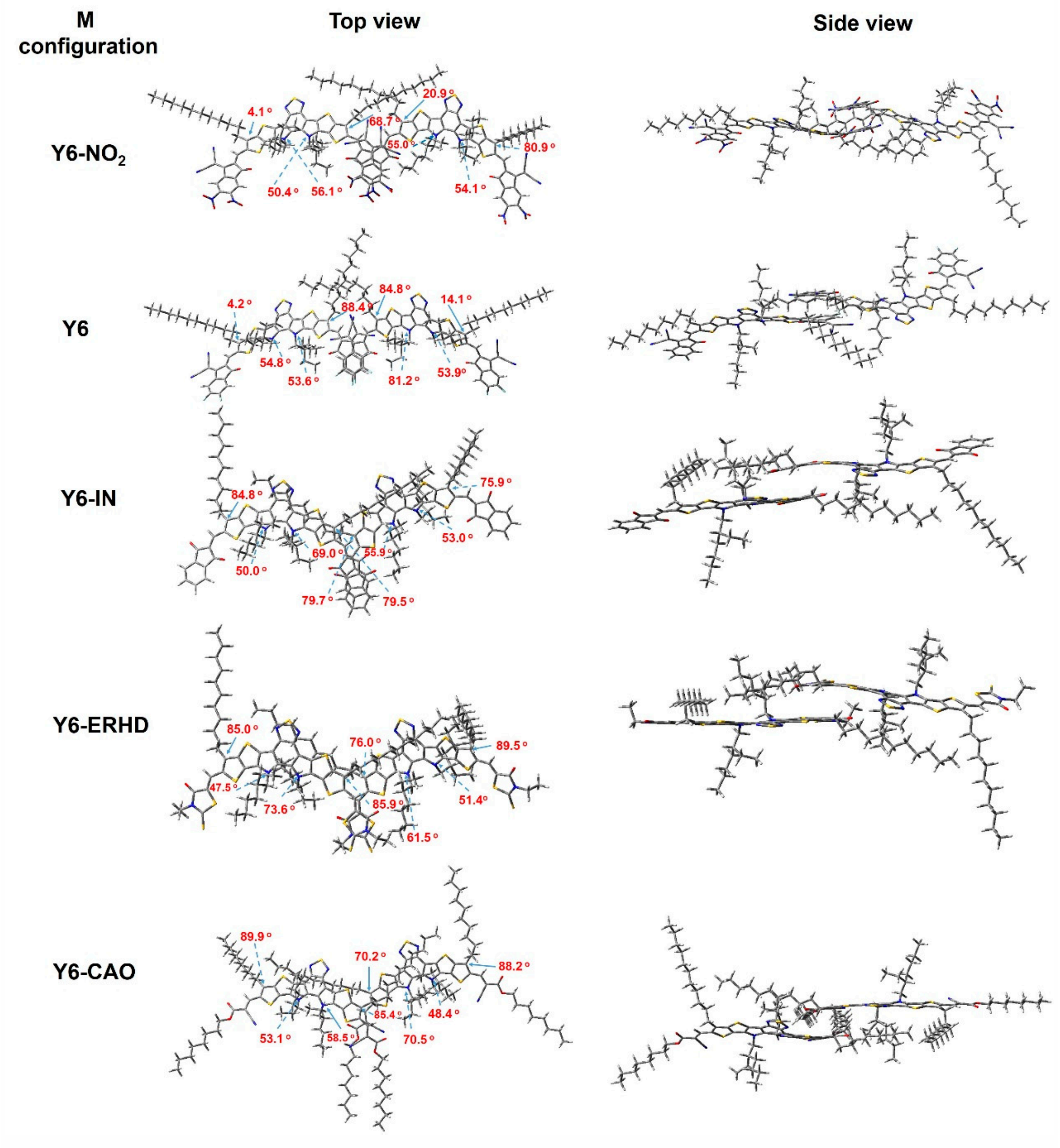

Figure S1. Dihedral angles of side chains of $Y6\text{-}NO_2$, Y6, Y6-IN, Y6-ERHD and Y6-CAO dimer with M configuration.

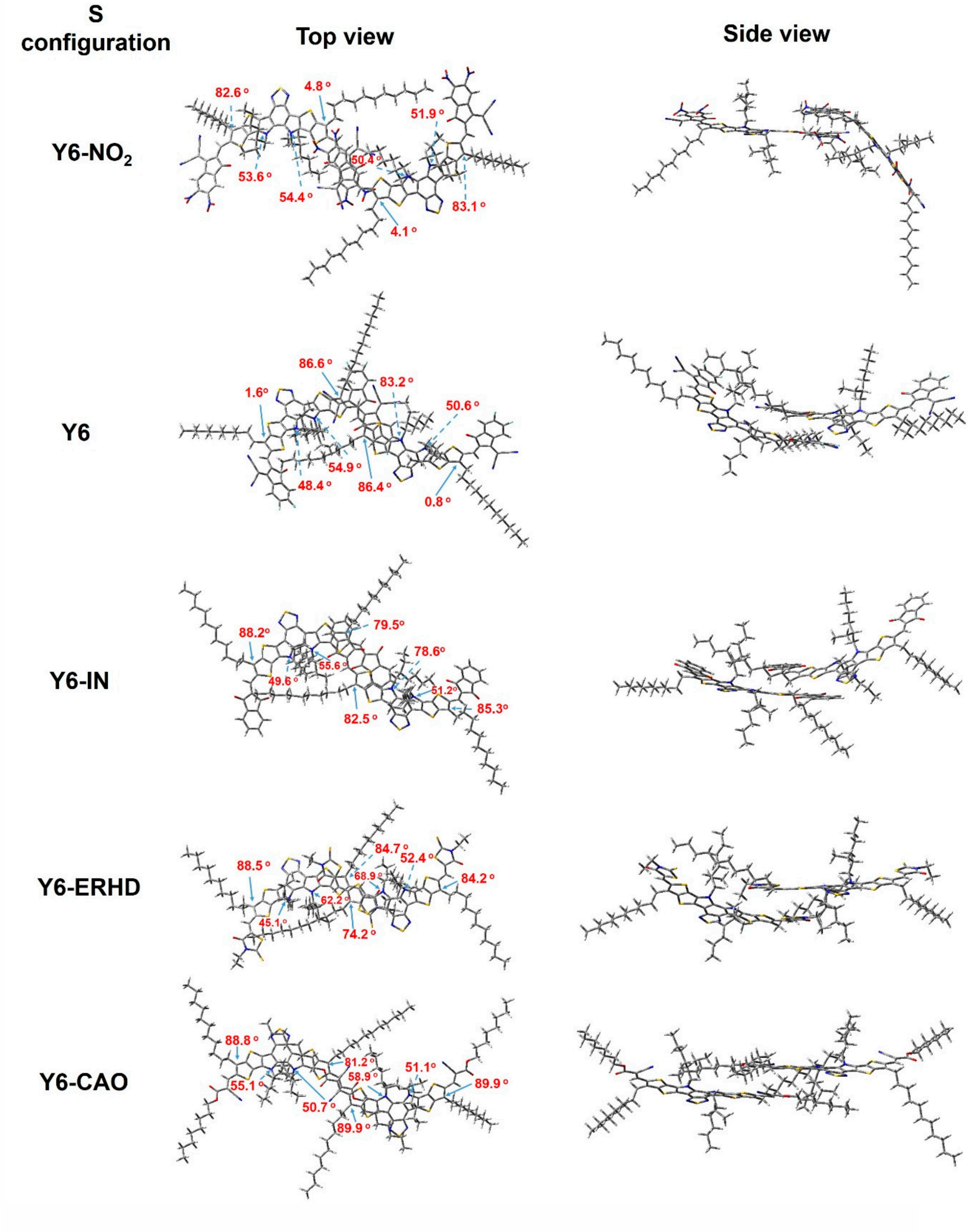

Figure S2. Dihedral angles of side chains of Y6-NO_2, Y6, Y6-IN, Y6-ERHD and Y6-CAO dimer with S configuration.

Table S4. Charge transfer distance (r, in Å), absolute value of electronic coupling ($|V_e|$ in meV), charge transfer rate constant (k_e, in s^{-1}), reorganization energy (λ, in eV) of electron transfer, charge carrier mobility (μ_e, in $cm^2\ V^{-1}\ s^{-1}$) of another dimer of Y6-NO_2, Y6, Y6-IN, Y6-ERHD and Y6-CAO.

	r	$\|V_e\|$	λ	k_e	μ_e
Y6-NO_2- S-dimer	17.73	8.11	0.33	7.91×10^{10}	4.80×10^{-2}
Y6-M-dimer	19.49	53.73	0.33	3.43×10^{12}	2.54×10^{0}
Y6-IN-S-dimer	14.93	10.59	0.37	8.83×10^{10}	3.80×10^{-2}
Y6-ERHD-S-dimer	13.62	9.56	0.34	9.34×10^{10}	3.40×10^{-2}
Y6-CAO-S-dimer	16.06	23.52	0.40	3.10×10^{11}	1.56×10^{-1}
